# The Chinese Question in Its Medical Bearing

**Published:** 1878-09

**Authors:** 


### EXCERPTA.

THE CHINESE QUESTION IN ITS MEDICAL BEARINGS.—It is always to be sincerely regretted when scientific men misuse facts to bolster political and social prejudice. We cannot but regard in this light some of the recent expressions of the California medical press in regard to the Chinese. Everybody knows that the jealousy of the rabble and the discreditable enmity of the "hoodlums" (*who have votes*), have so infected the political parties of that State, that hardly a man is to be found there honest and bold enough to stand up for the principles for which this free government was founded. They are all willing to deny and renounce the one great foundation principle of our Declaration of Independence and Constitution, at the beck and order of the lowest and narrowest class. The Christian religion and the natural freedom of man are declared to be unable to withstand the introduction of Mongolian emigrants!

That even medical men should cater to this nothing else than contemptible subserviency (or disgraceful cowardice), we did not expect; and were, therefore, painfully surprised to find the *Western Lancet*, in a long editorial, some months ago, trying to stir up and disseminate this prejudice, not only in California, but elsewhere in this country. It prints an appeal on the subject, which we give the benefit of circulation. After speaking of the number of prostitutes in San Francisco, "mostly young girls of the Mongolian race" (in passing, we add, that there is very positive authority for saying that the majority of prostitutes in San Francisco are *not* Mongolians,) and that, of course, syphilis is common, the writer proceeds:

"While the labor question may engage the attention of the community, legislators, and the nation at large, the subject of social evil, and the engrafting of formidable types of disease on our race, should occupy the attention of medical men.

"Leprosy is another of the gifts of Asiatic civilization to mankind, and are we to lie idle and allow this to be engrafted on the future generations that will occupy our places when we have paid the last debt to nature? We trust that the medical men of this State and the adjoining States will give these subjects their attention, and carefully follow up the suggestion, tracing to their origin many of the sad cases of necrosis, tubercle, paralysis in all its varied forms, and the multitude of hydra-headed marks that this foul disease entails on man when contracted in early life. Also, if there is not a specific difference in the type of disease which is introduced from the Mongolian race and that with which the Caucassian race has been affiicted for ages, under the rule of indiscriminate intercourse."

Now, *we trust* that this utterly groundless appeal to the passions and prejudices will meet with the condemnation it deserves. There is notoriously not the slightest basis for the assertion that "Mongolian syphilis" is one whit worse than the genuine native American article. The reports of the British Hospital, at Hong Kong, for many years past, are printed and easily accessible, and prove the falsity of the charge. The assertion that, because leprosy finds its historical origin in Asia, we should forbid all Asiatics our shores, shows the narrowness and worthlessness of the anti-Chinese arguments. Leprosy is, and has been, more prevalent in western than in eastern Asia. Especially severe has it been in Judea, whence, however, we have another "gift of Asiatic civilization," to-wit, the Bible, which teaches us the brotherhood of man, and specifically that, if a man is a leper or a woman a Magdalen, we should not, on that account, shut our doors to them and forbid them our presence. Yet, just this is what this specimen of American civilization calls upon the profession, whose mission is the healing art, to do.

The whole anti-Chinese agitation is an outcome of the worst and lowest enmities of selfish class strife; it has been fostered by narrow-minded or unscrupulous demagogues, in order to catch the votes of the masses; it has extended by a contagion of mistrust and bigotry far more to be feared than leprosy, to the cultivated, even to the philanthropic, few. Now that it is deliberately urged on the medical profession, this journal pronounces on the position it shall take in language of no uncertain ring. The Chinese ought to receive as warm a welcome throughout the whole of this broad land as the German, the Irishman, the Englishman, or the African. Our gates should be opened to them without restriction; his comings and goings should be aided and encouraged; the selfish classes who would abuse him, and the blinded men of education who berate and falsify about him, should alike be repressed—the one with the prompt and strong arm of the law, the other with the open and general condemnation of their enlightened compeers.—*The Medical and Surgical Reporter.*

